# Lysosome Dynamic Properties during Neuronal Stem Cell Differentiation Studied by Spatiotemporal Fluctuation Spectroscopy and Organelle Tracking

**DOI:** 10.3390/ijms21093397

**Published:** 2020-05-11

**Authors:** William Durso, Manuella Martins, Laura Marchetti, Federico Cremisi, Stefano Luin, Francesco Cardarelli

**Affiliations:** 1 NEST Laboratory—Scuola Normale Superiore, Piazza San Silvestro 12, 56127 Pisa, Italy; william.durso@sns.it; 2 Bio@SNS Laboratory—Scuola Normale Superiore, via G. Moruzzi, 1, 56126 Pisa, Italy; manuella.martins@sns.it (M.M.); federico.cremisi@sns.it (F.C.); 3 Center for Nanotechnology Innovation@NEST (CNI@NEST), Piazza San Silvestro 12, 56126 Pisa, Italy; laura.marchetti@unipi.it; 4 NEST, Istituto Nanoscienze, CNR, Piazza San Silvestro 12, 56127 Pisa, Italy

**Keywords:** lysosome, differentiation, neuronal stem cells, single-particle tracking, dynamics, fluctuation spectroscopy, *i*MSD

## Abstract

We investigated lysosome dynamics during neuronal stem cell (NSC) differentiation by two quantitative and complementary biophysical methods based on fluorescence: imaging-derived mean square displacement (*i*MSD) and single-particle tracking (SPT). The former extracts the average dynamics and size of the whole population of moving lysosomes directly from imaging, with no need to calculate single trajectories; the latter resolves the finest heterogeneities and dynamic features at the single-lysosome level, which are lost in the *i*MSD analysis. In brief, *i*MSD analysis reveals that, from a structural point of view, lysosomes decrement in size during NSC differentiation, from 1 μm average diameter in the embryonic cells to approximately 500 nm diameter in the fully differentiated cells. Concomitantly, *i*MSD analysis highlights modification of key dynamic parameters, such as the average local organelle diffusivity and anomalous coefficient, which may parallel cytoskeleton remodeling during the differentiation process. From average to local, SPT allows mapping heterogeneous dynamic responses of single lysosomes in different districts of the cells. For instance, a dramatic decrease of lysosomal transport in the soma is followed by a rapid increase of transport in the projections at specific time points during neuronal differentiation, an observation compatible with the hypothesis that lysosomal active mobilization shifts from the soma to the newborn projections. Our combined results provide new insight into the lysosome size and dynamics regulation throughout NSC differentiation, supporting new functions proposed for this organelle.

## 1. Introduction

Lysosomes and lysosome-related organelles, which comprise early and late endosomes, constitute a dynamic network of vesicles that traffic and process substrates coming from inside and outside the cell through the connection with autophagy and endocytosis processes, respectively. The array of acidic hydrolases in the lysosome endows this specific organelle with the ability to digest almost all the biomolecules delivered into it [1]. In light of its ubiquity and highly specialized function in all differentiated cells, it appears clear that defective biogenesis/maturation or malfunctioning of this organelle would negatively impact human health. A clear example of this is the lack of a single lysosomal enzyme in lysosomal storage disorders [2], which results in the abnormal accumulation of undigested substrates and lack of downstream products with severe consequences for neuronal cells in particular. Neurons are particularly dependent on lysosomal functions throughout the process of development and beyond, also given their longevity and exceptional polarization [3]. The spatial complexity of these cells, emerging during differentiation and imparted by their projections (axons and dendrites), requires fine regulation of lysosome structural (i.e., size) and dynamic properties throughout the cell lifespan. In particular, during neurite development and maintenance, vesicles and organelles need to be transported back and forth between different cell districts [4,5]; accordingly, a change in their type of dynamics (from mostly Brownian or confined to mostly drifted/superdiffusive) could be expected before or at the beginning of pivotal changes in these processes. For instance, active transport is switched on for lysosome movement towards their final destination within the abovementioned cellular domains. Lysosomes are transported to the neural projections along microtubules from the soma to the periphery, and vice versa, by means of molecular motors [6]. The retrograde transport in the axon is characterized by progressive acidification of the precursor vesicles (autophagosome and endosome) as they proceed from the axon terminal to the soma, a process called “axonal lysosome maturation” [7].

Many important parameters can be extracted from the analysis of the motion of vesicles inside cells [8,9,10,11]. The dynamics of vesicles can be explored by different methods within fluorescence (or, in general, optical) microscopy; amongst these, we selected imaging-derived mean squared displacement (or *i*MSD) and single-particle tracking (SPT). The former is a method based on the spatiotemporal correlation analysis of fluorescence fluctuation; it allows fast and robust extraction of the average dynamic features of moving objects (from molecules to entire organelles) directly from stacks of images, with no need of calculating the single trajectories [12,13,14]. The latter, through localization, makes it possible to retrieve information on single molecules or vesicles [10,15,16,17], which are averaged out by the *i*MSD method. The mean square displacement (MSD) calculated in both methods (averaged in a movie, in the former, and for each trajectory, in the latter) can be analyzed for extracting quantitative dynamics parameters and possibly their distribution and changes with time [18]. In particular, it is possible to infer a Brownian, superdiffusive, or subdiffusive trend of dynamics for the moving objects by analyzing the so-called ‘anomalous-diffusion coefficient’ (hereafter referred to as α coefficient), a parameter related to the shape of MSD(τ).

In this paper, we investigated the lysosome dynamics during the differentiation of neuronal stem cells (NSCs), obtained from mouse embryonic stem cells (mESCs) differentiated using a protocol that mimics cortical development in vitro [19]. Embryonic stem cells (ESCs) are pluripotent cells with the capacity to renew themselves or to differentiate into any of the three embryonic germ layers in vivo [20], and into the majority of cell types in vitro when subjected to the appropriate conditions. When mESCs are cultivated in chemically defined minimal medium (CDMM) and without external signals, they spontaneously undergo the entire process of neural differentiation from the very initial state [19,21,22]. Thus, the use of mouse ESC-derived NSCs allows setting up cultures of very early neural progenitor cells, which would be very difficult to dissect from embryos. In order to control the specific neuronal type, it is possible to exploit Wnt (wingless-related integration site), BMP (bone morphogenetic protein), SHH (sonic hedgehog), and FGF (fibroblast growth factor) signaling pathways [23], whose modulation is responsible for telencephalon regionalization in vivo [24]. In particular, inhibition of Wnt and BMP signaling pathways at early stages of development is essential for the specification of the forebrain, and this was exploited to obtain developing cortical neurons [19].

In this model, *i*MSD was used to quantitatively address the vast population of lysosomes in the cell soma during the differentiation process. This analysis revealed a decrement in size of lysosomes with time, and modification of key dynamic parameters, such as the average local organelle diffusivity and anomalous coefficient; these changes may parallel cytoskeleton remodeling during the differentiation process. SPT, on the other hand, allowed mapping heterogeneous dynamic responses of single lysosomes in different districts of the cells, and was the only analysis applied to movies done on projections, where only few organelles were imaged. SPT analysis highlighted a significant decrease of lysosomal transport in the soma followed by a rapid increase of transport in the projections at specific time points during neuronal differentiation, an observation compatible with the hypothesis that lysosomal active mobilization shifts from the soma to the newborn projections.

## 2. Results and Discussion

### 2.1. The Biological System

In this study, to analyze lysosome dynamics in cortical neurons, we differentiated mESCs using the protocol described in detail in [19] and in Materials and Methods. Briefly, we started from mESCs preconditioned with a medium containing leukemia inhibitory factor (LIF) and two inhibitors (2i) of MAPK/ERK and glycogen synthase kinase 3b (GSK3b) pathways; these conditions make ESCs express NANOG, which allows the transcription of pluripotent genes and creates a lenient chromatin structure.

**Figure 1:**
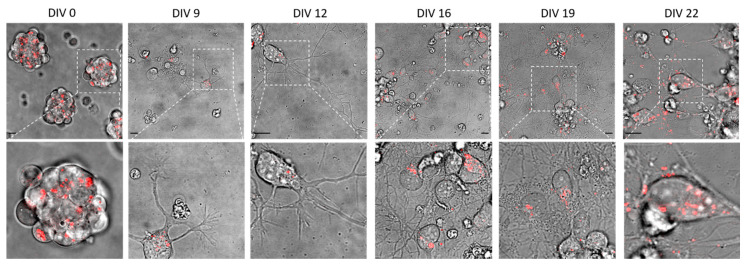
Microphotographs of living NSCs during differentiation, from DIV0 to DIV22. Bright-field microscopy images (**top**) with the respective magnification of the area selected by the dashed line (**bottom**). The bright field images are all superimposed to a confocal fluorescence microscopy image (a section within somas) of lysosomes labeled with LysoTracker Red (in red). Scale bars: 10 μm.

We were then able to mimic glutamatergic cortical neuron differentiation by administration of 53AH, a cyclohexyl analog of IWR-1 that selectively represses the Wnt pathway. Together with Wnt inhibition, LDN193189 hydrochloride was used as a BMP inhibitor working through ALK2/3 (type I receptor serine–threonine kinases) and SMAD pathway inhibition [25]. Representative microphotographs of living mESC-derived NSCs during differentiation, from days in vitro (DIV) 0 to DIV22, are reported in Figure 1. In the top line of images, and especially in the zoomed images in the middle line, it is possible to appreciate the progressive formation of a neurites network. The red channel in Figure 1, obtained by confocal fluorescence microscopy, shows how lysosomes can be observed thanks to labeling with LysoTracker Red.

### 2.2. iMSD Analysis of Lysosome Dynamics during NSC Differentiation

**Figure 2:**
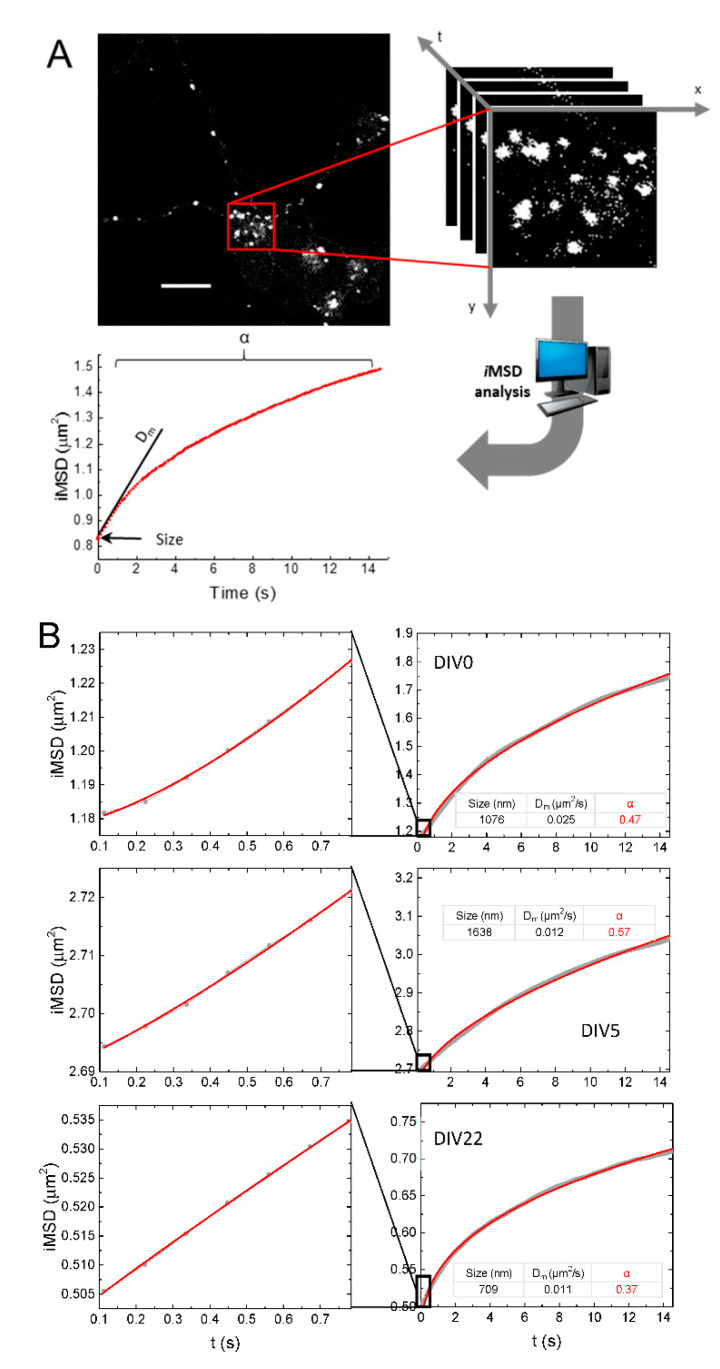
(**A**) *i*MSD analysis workflow. Top left: confocal fluorescence microscopy image of an NSC shows the distribution of lysosomes labeled with LysoTracker Red. Scale bar, 10 µm. Top right: a stack of images from a subregion of the image on the left, acquired with temporal resolution of 112 ms. Scale bar: 10 μm. Bottom: exemplary plot of iMSD (red trace) vs. time of NSC lysosomes populations, with a scheme of how the three output parameters (size, D_m_, and α) are determined. (**B**) Gray dots and lines: exemplary iMSD plots obtained from imaging of living cells stained with LysoTracker at DIV0, 5, and 22. Red: fits with a power law with offset. Right: long temporal scale (14.6 s). Left: short temporal scale (0.8 s).

In order to study lysosome dynamics during NSC differentiation by means of *i*MSD, we first fast-imaged a subcellular region of interest within the cytoplasm (Figure 2A,B). *i*MSD traces from each acquisition were obtained through spatiotemporal correlation analysis. *i*MSD fitting then provided information related to average lysosome structural and dynamic properties. In particular, we extracted three parameters: (i) size (the square root of σ_0_^2^, *y*-axis intercept of the *i*MSD trace), proportional to the average organelle diameter, as previously demonstrated [8]; (ii) D_m_, the local diffusivity, proportional to the initial slope of the *i*MSD; (iii) α, the anomalous diffusion coefficient (the exponent for a power-law fit, with offset, of the MSD), which has values close to, higher than, and lower than 1 for Brownian diffusion, superdiffusive, and subdiffusive or confined motion, respectively (Figure 2A). Differently from σ_0_^2^ and D_m_, the α coefficient can be calculated on different temporal windows depending on the timescale of interest. Here, in particular, we calculate the α coefficient on two timescales: 15 s, for calculating the overall α coefficient, and below 0.7 s, for calculating the “α (short t)”; the latter is particularly useful to highlight the peculiar superdiffusive motion of lysosomes at a short timescale, if present [8].

We checked that the extracted parameters arise only from organelle motion and are not affected by overall cellular movements. These latter, according to the theory of spatiotemporal image correlation spectroscopy [12,26], would appear as shifts in the position of the peak of the correlation function (which were not observed in our measurements). Graphs in Figure 2B show the *i*MSD plots from three exemplary movies at different neuronal differentiation times (DIV0, DIV5, and DIV22), and at two different time scales. As expected, at long time scale (14.6 s), *i*MSD graphs display an average subdiffusive motion for the lysosomal populations (α = 0.47 for DIV0, 0.57 for DIV5, and 0.34 for DIV22). On short time scale (0.8 s), *i*MSD plots reveal average superdiffusive or mostly Brownian behaviors for lysosomes (α = 1.4 for DIV0, 1.2 for DIV5, and 0.94 for DIV22 in the examples of Figure 2B). These results nicely match what we previously reported: a superdiffusive mode of motion emerges as a collective behavior at short spatiotemporal scales, while subdiffusion dominates at longer spatiotemporal scales [8].

**Figure 3:**
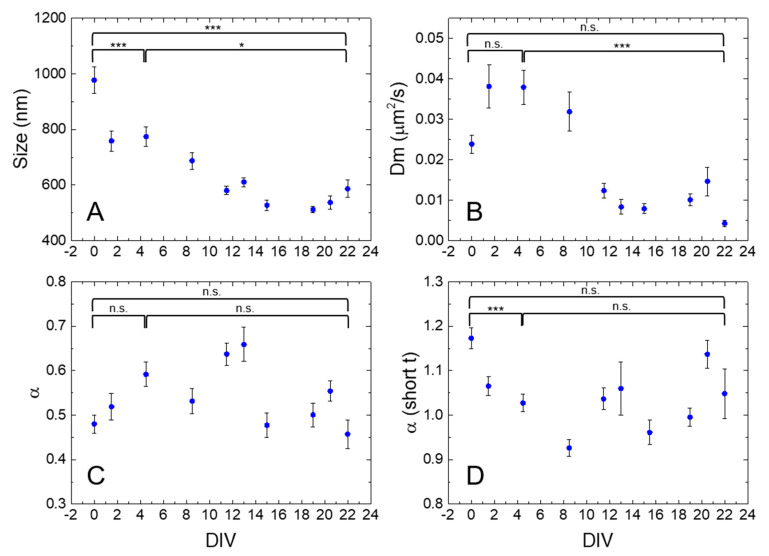
Plots of average size (**A**), D_m_ (**B**), α (**C**), and α at short times (**D**) values extracted from *i*MSD analysis of NSC lysosome populations acquired during differentiation (DIV0-22). Error bars: Standard Error (SE). The population means of different time points for size, D_m_, α, and α (short t) are all significantly different by one-way ANOVA (*p* < 0.0001). The results of the post hoc Tukey test are shown only for the comparisons amongst DIV0, 4.5, and 22; p-values: n.s. not significant, * <0.05; *** <0.001. Size, D_m_, α, and α (short t) average values with the respective DIV, N, and SE are reported in Table S1.

Figure 3 reports the average size, D_m_, and α coefficients extracted from *i*MSD plots of lysosomes inside the somas of NCSs throughout the entire neuronal differentiation process, and we show explicitly the statistical significance of the differences amongst only three exemplary time points of the process, DIV0, DIV4.5, and DIV22 (*post hoc* Tukey test for all the multiple comparisons). From a structural point of view, lysosomes displayed a significant decrement in size during neuronal stem cell maturation, from ~1 μm diameter at DIV0 to ~750 nm at DIV4.5 and, finally, to ~550 nm at the end of the differentiation protocol (DIV22) (Figure 3A).

Such a progressive reduction of lysosome diameter during neuronal development might be the result of endosome maturation into lysosome, a process that in the neuronal cell occurs mostly in axons [7]. The restricted diameter of the emerging neuronal projection may act as “size filter” for the endosomes’ access to the maturation process. Furthermore, the observation that presynaptic biogenesis requires axonal transport of lysosome-related vesicles [27] corroborates this hypothesis.

Lysosome dynamics are subjected to modification during neuronal maturation, particularly in terms of local diffusivity. After a transient increase during the early phases of differentiation (with a maximum at 0.04 μm^2^/s around DIV5), D_m_ significantly decreased down to a plateau value of ~0.01 μm^2^/s at DIV13-to-DIV22 (Figure 3B). As compared to D_m_, the anomalous coefficient was less affected during the process (Figure 3C). The transient increase of local diffusivity D_m_ and of anomalous coefficient α (even if the differences of α amongst DIV0, 4.5, and 22 were not significant after Tukey corrections) till DIV5 might reflect cytoskeleton remodeling towards a more organized structure [28]. Indeed, at DIV5, we observed a diffuse polymerization of actin filaments within the cells (Figure S1D–F). When the cytoskeleton reached a highly organized structure (DIV14, Figure S1G–I), the D_m_ dropped to lower values, indicating a confinement exerted by the bundles of actin filaments on the organelles. A similar role of actin has been reported for the regulation of membrane mobility of selected membrane receptors [15]. The standard error (SE) associated with both the size and the local diffusivity becomes sensibly smaller during differentiation, probably reflecting an ongoing process by which the highly heterogeneous (both in size and dynamics) population of lysosomes at DIV0 progressively becomes more homogeneous and acquires its final structural/dynamic identity in the fully differentiated cell.

The α-parameter at short time scale (for *t* < 0.8 s), after an initial decreases from a maximum value of 1.17 (DIV0, more superdiffusive behavior) in the first days of differentiation, had then a more scattered behavior around 1, the value characterizing a mostly diffusive motion (Figure 3D). The behavior of this parameter did not seem to reproduce the one observed for the previously discussed ones. However, it must be noted that this parameter can be calculated for most of the *i*MSD curves, also for the ones where the fit on the total curves is not appropriate (see number of observations in Table S1) because the *i*MSD has a non-monotonic (or not smooth enough) trend (see below).

**Figure 4:**
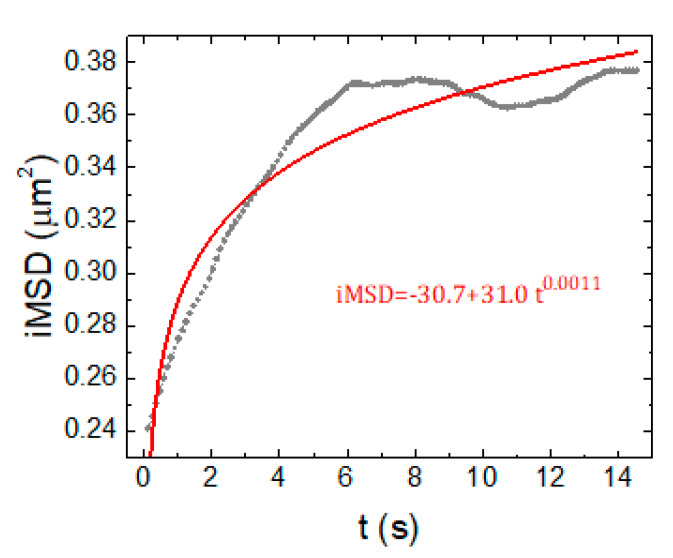
Representative *i*MSD plot of a neuronal projection at DIV12 (grey trace). Red trace: shifted-power fit with R^2^ = 0.91 and equation shown in red.

Of particular note, since we observed the neurite outgrow at DIV12, the acquisitions of lysosomes in the neuronal projections started from this time point. The analysis of lysosomes motion in the projections generated *i*MSD plots that did not correctly fit with the shifted power-law model we adopted to extract our parameters (R^2^ < 0.98 and parameters with values outside reasonable bonds at long time scales, see the example in Figure 4). This might be due to: (i) an intrinsic limitation of the *i*MSD technique when used to analyze series of images with a low number of moving objects; and (ii) the high variability in the motion type and parameters displayed by the lysosomes especially in those regions. Indeed, one can notice few fast-drifting vesicles amongst mostly blocked, confined, or slowly diffusing ones. Moreover, *i*MSD analysis is more influenced by the brighter vesicles in a movie, and we noticed that more confined vesicles were more often brighter, especially at later DIV and in the projections. In order to tackle more quantitatively these issues, we decided to apply a single-particle tracking (SPT) approach.

### 2.3. SPT Analysis of Lysosome Motion during NSC Differentiation

**Figure 5:**
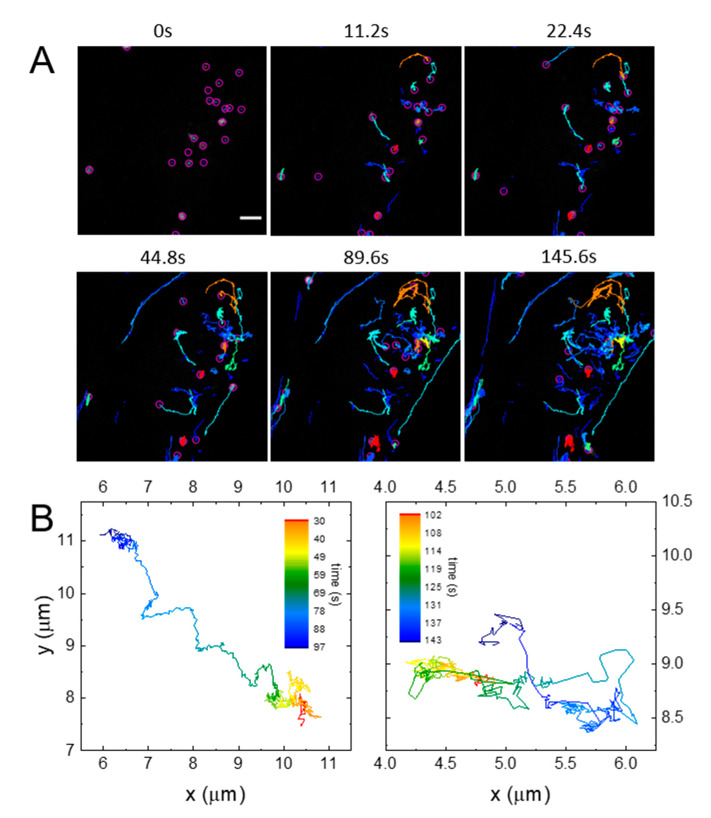
(**A**) Representative images from time-lapse video of moving lysosomes within a differentiating NSC. Each colored trace represents trajectories traveled by different lysosomes. Scale bar, 2 μm. (**B**) Exemplary lysosome trajectories at DIV5. The color of the trajectories scales with time.

To properly characterize lysosome dynamics in all cell districts (cell body and projections), trajectories were extracted from some of the same movies used for iMSD analysis (Figure 5). Figure 5A exemplifies the very different types of motions undergone by the lysosomes, as described above. A more careful analysis of the trajectories revealed that this variability existed within the trajectories of single lysosomes (Figure 5B). In order to extract the fraction of time that the vesicles spent undergoing different motion, it was necessary to separate each trajectory into subtrajectories with a single self-similar motion type.

**Figure 6:**
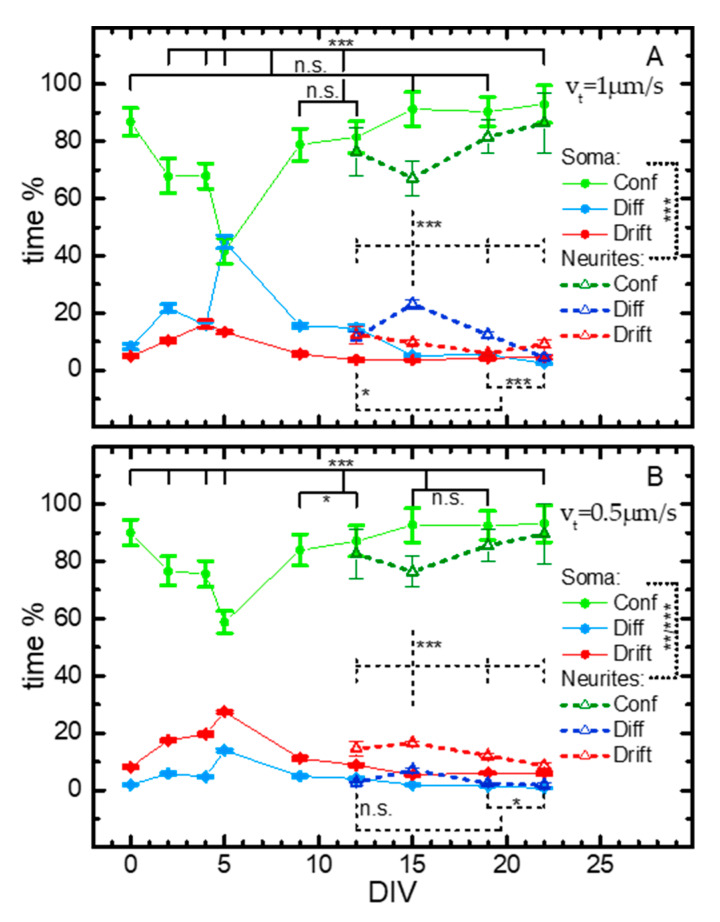
Dependence on DIV of the distributions (in %) for the time spent by lysosomes in confined (Conf), diffusive (Diff), and drifted (Drift) motion during neural differentiation, within cell bodies (DIV0-22, dots and solid lines) and projections (DIV12-22, triangles and dashed lines). Velocity threshold for drifted subtrajectories in the first analysis step (see text) v_t_ = 1.0 μm/s (**A**) and 0.5 μm/s (**B**). The differences at different DIVs were very highly significant (*p* < 10^−20^) within each case (soma or projections), according to a $\chi^{2}$ test (see Section 4 for tests and uncertainties). Distributions in soma and dendrites were highly significantly different at each considered DIV. (Bonferroni-adjusted) *p*-values for single comparisons: n.s. >0.05; * <0.05; ** <0.01; *** <0.0015. All the statistical data for these graphs are reported in Table S2

This was usually done in two steps: first, the longest, fastest, and most directional drifted subtrajectories were extracted, similar to what was done in [10]. Then, the remaining trajectories (or parts of trajectories) were controlled for transient arrest of diffusion (TAD), and the obtained subtrajectories were classified as fast diffusing, slow/confined or blocked, and drifted, according to the parameters characterizing their motion (amongst which were short-time diffusivity *D* and anomalous diffusion coefficient α as in Figure 2) as in [29,30]. The number of starting trajectories and obtained (sub)trajectories are reported in the Supplementary Table S2. The times that the lysosomes spent undergoing the different kinds of motion were calculated by summing the duration of the various (sub)trajectories classified as above described. Figure 6 reports these results across nine time points, from the embryonic stem cell stage (DIV0) to the mature neuronal cell (DIV22), and considering two velocity thresholds for drifted subtrajectories in the first analysis step (v_t_): v_t_ = 1.0 µm/s, similar to the one chosen in [10], and v_t_ = 0.5 µm/s (Figure 6A,B, respectively). The difference between the two graphs is due to the peculiar vesicle dynamics, as explained later. Lysosome motion was measured in cell bodies at all time points in the differentiation process, and from DIV12 to DIV22 in neuronal projections because they were not evident up to DIV9. In cell bodies, the early differentiation stage was characterized by an increase of both drifted and diffusive relative components, which reached a maximum at DIV5, with a consequent minimum in the “confined” category. After four days (DIV9), both drifted and diffusive relative fractions decreased and remained low to the last time point. Plots based on the evaluation with v_t_ 1.0 µm/s and 0.5 µm/s (Figure 6A,B, respectively) display similar behaviors, but with a usually higher impact of diffusive components for v_t_ = 1.0 µm/s. For example, at DIV5, diffusive motion was undergone for 45% of the time, while drifted and confined ones for 15% and 35%, respectively; the same percentages were 14%, 27%, and 59% (diffusive, drifted, and confined, respectively) when v_t_ = 0.5 µm/s, instead. This can be explained by looking closely at a trajectory considered “diffusive”, especially for v_t_ = 1.0 µm/s: often, it appeared as composed of more slow or confined pieces connected by more drifted ones (Figure 5B, right), which were, however, too short or too slow to be recognized by either analysis step. Decreasing v_t_ allowed recognizing more of these parts, therefore the (sub)trajectories initially considered diffusive were divided into confined and drifted subtrajectories.

In the projections, we can notice a similar behavior to that in the soma compartments, but with less pronounced changes and delayed by ~10 days: drifted and diffusive relative fractions transiently increased with maximum at DIV15 and, after seven days (DIV22), they decayed to the starting value (Figure 6, empty symbols).

Overall, data coming from SPT analysis confirm that most of the analyzed lysosomes, both in the soma and in the projections, displayed a confined mode of motion regardless of the DIV investigated. This is line with the iMSD data reported in Figure 2B showing an average subdiffusive dynamic behavior for the whole organelle population. However, we also unveiled that lysosomal active transport within the cell body had a transient but substantial increase at DIV5, which shows an intriguing correspondence in time to the increase in expression of the neural progenitor marker *Sox1*, as measured by some of us [19] and others [31,32,33]. Then, a dramatic decrease of lysosomal transport in the soma (DIV9) was followed by a rapid increase of transport activation of lysosomes in the projections at DIV15. The latter could be less pronounced than in the soma, as a result of not perfect synchronization between the outgrowth of different neurite processes. Our observations are compatible with the hypothesis that lysosomal active mobilization shifts from the soma to the newborn projections. Interestingly, a distal accumulation of signaling endosomes transporting neurotrophic factors was recently found in the elongating axons of neurons [11]. It is thus tempting to hypothesize that the distal mobilization of lysosomes may as well support the elongation process, possibly contributing to presynaptic biogenesis [27].

## 3. Conclusions

In our work, we studied lysosome dynamics during NSC differentiation using two different but complementary approaches: a population-based method (*i*MSD) and a single-object method (SPT). Our results provide new insight into the lysosome dynamics throughout NSC differentiation, supporting new functions proposed for this organelle and opening to new studies on the pathophysiological role of lysosomes and lysosome-related organelles in human health and disease. The differential activation of lysosomal transport between cell body and projection matches the emerging idea of the lysosome as a signaling hub [34]. Recent evidence, furthermore, suggests that lysosomes might participate in local axonal translation [35]: mRNA granules, in fact, hitchhike on lysosomes for axonal transport [36]. Therefore, a better comprehension of lysosomal biophysics could shed new light on the relationship between gene expression and synaptic activity.

## 4. Materials and Methods

### 4.1. Mouse ES Cell-Derived Neural Cell Culture

Murine ES cell lines E14Tg2A (pp. 35–40) were cultured and neuralized essentially as described [19], with minor modifications. For ES cell expansion, cells were grown on gelatin-coated tissue culture dishes (pretreated 10′ with 0.1% gelatin in PBS) at a density of 40,000 cells/cm^2^. ES cell medium, changed daily, contained GMEM (G5154, Sigma-Aldrich, St. Louis, MI, USA), 10% fetal calf serum (12133C, Sigma-Aldrich, St. Louis, MI, USA; FCS), 2 mM glutamine (25030, Thermo Fisher Scientific, Waltham, MA, USA), 1 mM sodium pyruvate (25030, Thermo Fisher Scientific), 1 mM nonessential amino acids (NEAAs, 11140, Sigma Aldrich), 0.05 mM β-mercaptoethanol (M3148, Sigma Aldrich), 100 U/mL penicillin/streptomycin (15140, Thermo Fisher Scientific), and 1000 U/mL recombinant mouse LIF (PMC9484, Thermo Fisher Scientific). Before starting the differentiation protocol, cells were passaged three times in 2i + LIF medium, in which FCS was substituted by two chemicals: PD0325901 (Sigma) and CHIR99021 (Sigma), which work as two inhibitors (2i) of MAPK/ERK and glycogen synthase kinase 3b (GSK3b) pathways, respectively. Chemically defined minimal medium (CDMM) for neural induction consisted of DMEM/F12 (21331-046, Thermo Fisher Scientific), 2 mM glutamine, 1 mM sodium pyruvate, 0.1 Mm NEAAs, 0.05 mM β-mercaptoethanol, 100 U/mL penicillin/streptomycin supplemented with N-2 supplement 100X (175020, Thermo Fisher Scientific), and B-27 supplement minus vitamin A 50X (125870, Thermo Fisher Scientific). The protocol of ES neuralization consisted of three steps. In the first step, dissociated ES cells were washed with DMEM/F12, seeded on gelatin-coated culture dishes (65,000 cells per cm^2^), and kept for 24 h in 2i + LIF medium. The day after (DIV0), medium was changed to CDMM plus 2.5 μM 53AH Wnt inhibitor (C5324-10, Cellagen Technology, San Diego, CA, USA) and 0.25 μM of the BMP inhibitor LDN193189 hydrochloride (SML0559, Sigma Aldrich), for three days, until DIV3. At this point (start of Step 2), ES cells were dissociated and seeded (65,000 cells per cm^2^) on poly-ornithine (P3655 Sigma-Aldrich; 20 μg/mL in sterile water, 24 h coating at 37 °C) and natural mouse laminin (23017015, Thermo Fisher Scientific; 2.5 μg/mL in PBS, 24 h coating at 37 °C). Cells were cultured for four additional days (until DIV7) in CDMM Plus Wnt/BMP inhibitors, changing the medium daily. Serum employed for Trypsin inactivation was carefully removed by several washes in DMEM/F12. At DIV7, cells were dissociated and seeded (125,000 cells per cm^2^) on poly-ornithine and laminin-coated wells. Subsequently, isocortical cultures were kept in CDMM Plus Wnt/BMP inhibitors from DIV7 to DIV22 (Step 3). On the 11th day of Step 3, DMEM/F12 was replaced with Neurobasal B27 and NEAAs were removed from the CDMM to avoid glutamate-induced excitotoxicity. Medium was changed daily until the next step.

### 4.2. Cell Fluorescence Staining

Cells prepared for fluorescence staining experiments were cultured on poly-ornithine/laminin-coated round glass coverslips. Cells were fixed using 2% paraformaldehyde for 12 min, washed twice with PBS, permeabilized using 0.1% Triton X100 in PBS, and blocked using 0.5% BSA in PBS for 1 h at RT. F-actin was stained incubating Alexa Fluor™ 647 Phalloidin (A22287, Thermo Fisher Scientific) 1:40, for 1 h at RT in PBS containing 1% BSA, followed by three PBS washes (10′ each). Nuclear staining was obtained with DAPI (D1306, Thermo Fisher Scientific). Cells were coverslipped with Aqua Poly/Mount (18606-100, Polysciences, Warrington, PA, USA).

### 4.3. Live-Cell Imaging

Cells prepared for live fluorescence imaging were seeded (at DIV-1, DIV3, or DIV7 depending on the DIV of measurement) in WillCo plates; they were incubated with LysoTracker^®^ Red DND-99, according to the manufacturer’s protocol, at final concentration of 60 nM, 30 min before microscopy acquisition. Confocal fluorescence image series were acquired with a Leica SP5 confocal microscope with a 100× NA 1.30 oil immersion objective. All experiments were carried out at 37 °C and 5% CO_2_ using an incubation chamber enclosing the microscope stage and body and a smaller chamber around the sample for CO_2_ control. A 561 nm DPSS laser was used to excite LysoTracker^®^ Red DND-99. Fluorescence emission was collected in the 570–670 nm range, with the PMT detector working in analog mode. The diameter of the detection pinhole was set to the standard size of 1 Airy. Sequential image series of 1300 frames at 8 bits were collected at a fixed pixel size of 67.5 nm selecting a region of interest of 256 × 256 pixels. The scan speed was set to the frequency of 1400 Hz, with a resulting frame time interval of 112 ms. The overall acquisition time was of approximately 145 s. All the acquisitions were grouped in 10 representative time points from day in vitro (DIV) 0 to 22 for the *i*MSD analysis (Table S1).

### 4.4. Image Processing and Data Analysis

The iMSD processing of the acquired image stacks and the subsequent data analysis were carried out with custom scripts in MATLAB (MathWorks Inc., Natick, MA, USA), as described in detail in Refs. [8,13]. For the parameter α at short time scale, the fits had been done for the first seven points of the iMSD, and the results were considered valid only when the anomalous diffusion fit was better, with a confidence level above 95%, than a fit with a constant (F-test, with *p*-value of 0.05). One-way ANOVA test was performed in order to evaluate if the population means of different time points for size, D_m_, α, and α (short t) were significantly different. Multiple comparisons were conducted using the *post hoc* Tukey test.

For the particle identification and the trajectory evaluation, we adopted TrackMate, a package in an open-source image-processing software (i.e., ImageJ), which provides the tools to perform single-particle tracking (SPT). The adopted detector for the particle identification uses the Laplacian of Gaussians (LoG) approach and the tracking algorithm is based on a linear assignment problem (LAP), which allows dealing with gap-closing events. The settings were optimized without considering splitting and merging events. Different MATLAB scripts were used to perform two consecutive analyses on the SPT-derived trajectories. The first step, fully described in [10], was based on the lysosome velocity (v) within a moving time-window of five frames: after a segmentation phase, trajectories or subtrajectories with v above a definite velocity threshold (v_t_) were considered as “drifted” (“go” motion as described in [10]). Other different parameters used here with respect to [10] were: minimum length for considering a trajectory: 15 frames; minimum length for subtrajectories, 4 frames; no minimum shift or average velocity for including a trajectory in the analysis. The nondrifted (sub)trajectories, with average v below v_t_, were analyzed by means of the moment scaling spectrum (MSS) and transient arrest of diffusion (TAD) as in [29,30]. This analysis allows further characterizing the organelle dynamics by quantifying the time spent by lysosomes in confined, diffusive, or drifted motion. Within the “confined” category were included all the motions classified as “immobile”, “slow”, and “TAD events” in [30]; the “drifted” category included the “drifted” subtrajectories as determined by both analysis steps. Parameters for TAD analysis different than in [30] were: time window of maximum *S_m_* = 12 steps, probability level threshold *L_c_* = 2.0, minimal duration *t_c_* of 6 steps for a transient confinement zone (TCZ); obtained (sub)trajectories were classified as in [30], with parameters optimized on the basis of careful inspection of present results in cell bodies and projections. The distributions (normalized to 100%) for time spent by lysosomes undergoing the various types of motion, and the relative uncertainties, were calculated similarly as in [17], where also the performed statistical test is described. Briefly, for each bin j (confined, diffusive, drifted), we summed the duration $t_{ji}$ of each of the $n_{j}$ (sub)trajectories i classified within j: the total time $f_{j}$ and its variance $\sigma_{f_{j}}^{2}$ were calculated as $f_{j}=\sum_{i=1}^{n_{j}}t_{ji}$, $\sigma_{f_{j}}^{2}=\frac{n_{j}}{n_{j}-1}\left(\sum_{i=1}^{n_{j}}t_{ji}^{2}-\frac{{\left(\sum_{i=1}^{n_{j}}t_{ji}\right)}^{2}}{n_{j}}\right)$. Differences were analyzed for significance using $\chi^{2}$ tests, with the statistic $\chi^{2}=\sum_{k=1}^{N_{p}}\sum_{j=1}^{N}\frac{{\left(f_{j}^{\left(k\right)}-E_{j}^{\left(k\right)}\right)}^{2}}{\sigma_{E_{j}^{\left(k\right)}}^{2}}$ (k refers to the $N_{p}$ compared distributions, $E_{j}^{\left(k\right)}$ is the expected value in the bin j with variance $\sigma_{E_{j}^{\left(k\right)}}^{2}$, N is the number of bins). This statistic follows a $\chi^{2}$-distribution with $\left(N_{p}-1\right)\left(N-1\right)$ degrees of freedom. $E_{j}^{\left(k\right)}$ and $\sigma_{E_{j}^{\left(k\right)}}^{2}$ can be calculated by pooling together the data from the $N_{p}$ populations.

## Supplementary Materials

The following are available online at https://www.mdpi.com/1422-0067/21/9/3397/s1, Figure S1: Confocal images showing F-actin stained with Alexa Fluor™ 647 Phalloidin, Table S1: Average values of size, D_m_, α and α at short times (α short) extracted from *i*MSD analysis of lysosomes during NSCs differentiation, Table S2: Statistical data for Figure 6.
