# Supplementary material for: Lysosome Dynamic Properties during Neuronal Stem Cell Differentiation Studied by Spatiotemporal Fluctuation Spectroscopy and Organelle Tracking
Source: Int J Mol Sci. 2020 May 11;21(9):3397. doi: 10.3390/ijms21093397 (PMC7247004; doi:10.3390/ijms21093397)
Supplement: Supplementary file 1 [file ijms-21-03397-s001.pdf]

Supplementary Tables

Table 1S. Average values of size, Dm, α and α at short times (α short) extracted from *i*MSD analysis of lysosomes during NSCs differentiation (DIV: day in vitro; SE: standard error).

Size			
DIV	N total	Mean	SE
0	39	978	48
1, 2	48	759	36
4, 5	80	774	34
8, 9	44	688	30
11, 12	74	581	15
13	35	611	16
15, 16	27	527	18
19	70	512	11
20, 21	22	538	23
22	18	587	31

D_m			
DIV	N total	Mean	SE
0	39	0.024	0.002
1, 2	48	0.038	0.005
4, 5	80	0.038	0.004
8, 9	44	0.032	0.005
11, 12	74	0.012	0.002
13	35	0.008	0.002
15, 16	27	0.008	0.001
19	70	0.010	0.002
20, 21	22	0.015	0.004
22	18	0.004	0.001

α			
DIV	N total	Mean	SE
0	39	0.48	0.02
1, 2	48	0.52	0.03
4, 5	80	0.59	0.03
8, 9	44	0.53	0.03
11, 12	74	0.64	0.03
13	35	0.66	0.04
15, 16	27	0.48	0.03
19	70	0.50	0.03
20, 21	22	0.55	0.02
22	18	0.46	0.03

α short			
DIV	N total	Mean	SE
0	74	1.17	0.02
1, 2	68	1.07	0.02
4, 5, 6	111	1.03	0.02
8, 9	70	0.93	0.02
11, 12	97	1.04	0.02
13	33	1.06	0.06
15, 16	52	0.96	0.03
19	93	1.00	0.02
20, 21	29	1.14	0.03
22	21	1.05	0.06

Table S2 (next page) Statistical data for Fig. 6. The total duration (time) of trajectories classified as undergoing a distinct type of motion (Conf: confined; Diff: diffusive; Drift: drifted) are reported for all observed DIV (days in vitro) in somas (soma) and projections (proj.), together with their uncertainties (Δ time) and their percentages (%) with respect to the reported totals (TOT). We report also the starting number of trajectories (trajs) and the final number of trajectories and/or subtrajectories [(sub)trajs] after the two steps of trajectory segmentation; the comparison of these numbers can give an idea of the heterogeneity of motion amongst and within single lysosome trajectories. v_t: velocity threshold in the first step of trajectory segmentation (see main text).

			v_t=1.0 μm/s					v_t=0.5 μm/s				
sample	trajs	type	time (s)	Δ time (s)	time %	Δ time %	(sub)trajs	time (s)	Δ time (s)	time %	Δ time %	(sub)trajs
DIV0	952	Conf	14609	795	86.8	4.7	966	15109	771	90.0	4.6	1182
		Diff	1392	167	8.3	1.0	198	316	21	1.9	0.1	124
		Drift	821	74	4.9	0.4	357	1359	73	8.1	0.4	1258
		TOT	16823	816	100.0	4.8	1521	16785	775	100.0	4.6	2564
DIV2	595	Conf	4600	407	67.9	6.0	477	5151	342	76.7	5.1	900
		Diff	1472	93	21.7	1.4	255	394	25	5.9	0.4	176
		Drift	704	45	10.4	0.7	493	1174	30	17.5	0.5	1422
		TOT	6776	420	100.0	6.2	1225	6719	345	100.0	5.1	2498
DIV4	967	Conf	4057	265	67.9	4.4	776	4500	269	75.6	4.5	1011
		Diff	947	62	15.8	1.0	243	284	21	4.8	0.3	142
		Drift	969	70	16.2	1.2	617	1169	37	19.6	0.6	1212
		TOT	5973	281	100.0	4.7	1636	5953	272	100.0	4.6	2365
DIV5	607	Conf	2998	317	41.6	4.4	539	4216	279	58.7	3.9	1081
		Diff	3236	159	44.9	2.2	570	999	39	13.9	0.5	452
		Drift	967	38	13.4	0.5	988	1968	33	27.4	0.5	2576
		TOT	7202	356	100.0	4.9	2097	7183	283	100.0	3.9	4109
DIV9	766	Conf	10797	775	78.9	5.7	762	11452	740	83.9	5.4	1097
		Diff	2118	114	15.5	0.8	339	672	47	4.9	0.3	233
		Drift	775	80	5.7	0.6	516	1519	82	11.1	0.6	1642
		TOT	13690	787	100.0	5.8	1617	13644	746	100.0	5.5	2972
DIV12 (soma)	680	Conf	10629	723	81.5	5.5	892	11321	708	87.1	5.4	1048
		Diff	1940	129	14.9	1.0	274	534	35	4.1	0.3	176
		Drift	478	66	3.7	0.5	302	1146	55	8.8	0.4	1264
		TOT	13047	738	100.0	5.7	1468	13001	711	100.0	5.5	2488
DIV15 (soma)	699	Conf	11647	762	91.3	6.0	670	11822	761	92.7	6.0	826
		Diff	661	63	5.2	0.5	114	248	25	1.9	0.2	80
		Drift	454	36	3.6	0.3	214	686	33	5.4	0.3	683
		TOT	12762	765	100.0	6.0	998	12756	762	100.0	6.0	1589
DIV19 (soma)	816	Conf	18970	1089	90.4	5.2	967	19389	1051	92.5	5.0	1165
		Diff	1140	89	5.4	0.4	163	318	29	1.5	0.1	108
		Drift	884	94	4.2	0.4	427	1258	65	6.0	0.3	1234
		TOT	20994	1097	100.0	5.2	1557	20965	1053	100.0	5.0	2507
DIV22 (soma)	635	Conf	12724	891	93.0	6.5	700	12715	890	93.1	6.5	721
		Diff	329	28	2.4	0.2	91	109	13	0.8	0.1	55
		Drift	628	79	4.6	0.6	241	827	80	6.1	0.6	536
		TOT	13681	895	100.0	6.5	1032	13651	893	100.0	6.5	1312
DIV12 (proj.)	183	Conf	3903	435	76.3	8.5	372	4208	439	82.6	8.6	359
		Diff	582	108	11.4	2.1	68	141	15	2.8	0.3	57
		Drift	633	154	12.4	3.0	204	746	136	14.6	2.7	527
		TOT	5118	474	100.0	9.3	644	5094	460	100.0	9.0	943
DIV15 (proj.)	481	Conf	6229	559	67.1	6.0	555	7060	493	76.4	5.3	1015
		Diff	2149	154	23.2	1.7	271	659	54	7.1	0.6	189
		Drift	906	63	9.8	0.7	584	1527	57	16.5	0.6	1566
		TOT	9284	583	100.0	6.3	1410	9246	499	100.0	5.4	2770
DIV19 (proj.)	551	Conf	11474	830	81.6	5.9	771	11973	781	85.5	5.6	1181
		Diff	1758	101	12.5	0.7	277	337	27	2.4	0.2	134
		Drift	837	44	5.9	0.3	669	1691	81	12.1	0.6	1786
		TOT	14069	837	100.0	5.9	1717	14001	786	100.0	5.6	3101
DIV22 (proj.)	205	Conf	3977	491	86.4	10.7	261	4117	482	89.5	10.5	271
		Diff	208	34	4.5	0.7	47	84	28	1.8	0.6	27
		Drift	419	68	9.1	1.5	142	397	46	8.6	1.0	315
		TOT	4604	497	100.0	10.8	450	4598	485	100.0	10.6	613

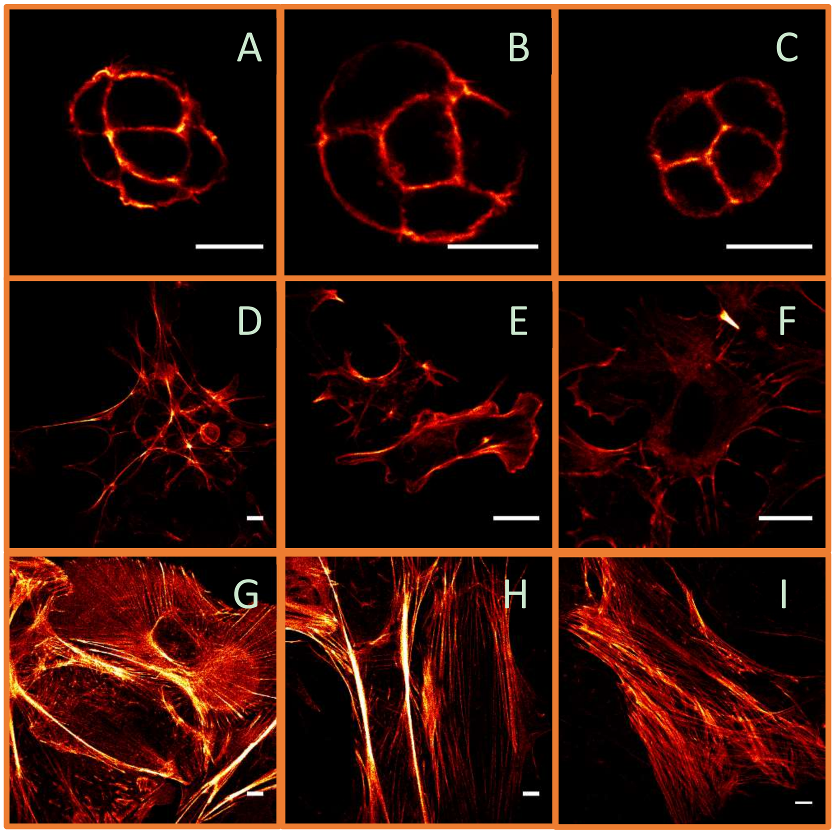

Figure S1. Confocal images showing F-actin stained with Alexa Fluor™ 647 Phalloidin. (**A, B, C**) DIV0 mouse embryonic stem cells (mESCs), at the beginning of cortical differentiation in medium without 2i and LIF. (**D, E, F**) Representative image of DIV5 mouse progenitor cortical neurons obtained by Wnt and BMP signalling pathways blockade in N2B27 medium. (**G, H, I**) Representative images of DIV14 mouse cortical projection neurons maintained in neurobasal B27 medium. Scale bars in all images, 10 μm.
